# Impaired learning from regret and disappointment in alcohol use disorder

**DOI:** 10.1038/s41598-020-68942-y

**Published:** 2020-07-21

**Authors:** Caterina Galandra, Chiara Crespi, Gianpaolo Basso, Nicola Canessa

**Affiliations:** 1 Istituti Clinici Scientifici Maugeri IRCCS, Cognitive Neuroscience Laboratory, 27100 Pavia, Italy; 2 Department of Brain and Behavioral Sciences, University of Pavia, Piazza Botta 6, 27100 Pavia, Italy; 3 University of Milano-Bicocca, 20126 Milan, Italy; 4 NEtS Center, Scuola Universitaria Superiore IUSS Pavia, 27100 Pavia, Italy

**Keywords:** Decision, Human behaviour, Addiction

## Abstract

The development of alcohol habits is considered a form of maladaptive reinforced learning, with sustained alcohol use resulting in the strengthening of associative links between consumption and either rewarding, or the lack of aversive, experiences. Despite recent efforts in characterizing decision-making skills in alcohol-use-disorder (AUD), it is still unknown whether impaired behavioural learning in AUD patients reflects a defective processing and anticipation of choice-related, cognitively mediated, emotions such as regret or relief for *what might have been under a different choice*. We administered a Wheel-of-Fortune (WoF) task to 26 AUD patients and 19 healthy controls, to investigate possible alterations in adjusting choices to the magnitude of experienced regret/relief, and in other facets of decision-making performance such as choice latency. AUD patients displayed both longer deliberation time than healthy controls, and impaired adaptations to previous outcome-related negative emotions. Although further evidence is needed to unveil the cognitive mechanisms underlying AUD patients’ abnormal choice, the present results highlight important implications for the clinical practice, e.g. in terms of cognitive treatments aiming to shape faulty perceptions about negative emotions associated with excessive alcohol exposure.

## Introduction

Decision-making involves several cognitive processes underlying the selection of the optimal choice among the existing alternatives^1,2^, including the assessment of risk when their potential outcomes are probabilistic rather than certain^3^. Considerable neurobiological evidence shows a close relationship between the computational and emotional facets of decision-making under risk, because the evaluation of prospective outcomes entails the anticipation of their rewarding or punishing affective consequences^4,5^.

Emotions are indeed considered to potentiate the appetitive or aversive drives generated by such anticipatory processes, thus modulating choice behaviour and adaptive behavioural learning^6–8^. In particular, Mellers’^9^ *Decision affect theory* emphasized the role of positive or negative affective states such as satisfaction or disappointment for outcomes better or worse than expected, respectively^10,11^. These basic feelings do not entail a sense of responsibility for probabilistic outcomes occurring regardless of one’s own decisions. Outcome evaluation and subsequent choices, however, are also shaped by the awareness that things might have been better or worse under a different choice, i.e. by the experience and anticipation of relief or regret, respectively^9^. These complex, cognitively mediated, emotions result from spontaneous counterfactual comparisons between the outcomes of selected vs. rejected options^12^, promoting the avoidance of the aversive experience of regret in subsequent choices^7,8,13–15^. The salience of such comparisons is enhanced both by the feeling of responsibility for one’s own outcomes^16^ and by the ease with which an alternative outcome can be mentally represented. The closeness between actual and counterfactual outcomes, resulting in so-called near-miss outcomes^17^, potentiates the affective and behavioural impact of regret-based learning^18^. The affective salience of near-miss outcomes is also considered to potentiate motivations towards maladaptive behaviours^19^ and might thus contribute to abnormal choices in pathological conditions. Indeed, the development of computational models of choice-related affects boosted the investigation of impaired decision-making in different neuro-psychiatric diseases, such as Parkinson’s disease^20^, obsessive–compulsive disorder^21^ or depression^22^. In particular, altered decision-making under risk represents a core phenotype in addictions, including alcohol use disorder (AUD)^23,24^.

Neurobiological models of addiction suggest that decision-making impairments in AUD may reflect defective behavioural adaptations to changes in reward contingencies, i.e. to “reward prediction errors” coding the difference between expected and actual outcomes^25^. The formation of alcohol habits is considered a result of maladaptive reinforced learning, strengthening the association between consumption and either rewarding, or the lack of aversive experiences^26^. The driving role of negative reinforcement, i.e., the need to escape the aversive state associated with the craving for alcohol^27^, might thus be increased both by the experience of regret, computationally coded as a “fictive prediction error”^25^, and by near-miss outcomes^19^. However, only few studies have investigated regret processing and/or anticipation in pathological populations^28,29^, including problem gambling^30^ but not AUD.

On this basis, we investigated possible alterations in adjusting choices to experienced disappointment, regret and near-miss outcomes in AUD patients compared with healthy controls. We used a Wheel of Fortune (WoF) task^31^, that allows to assess the extent to which choice behaviour is influenced by these variables, in addition to expected value^8^. In this task, subjects are repeatedly asked to choose between two gambles, depicted as wheels of fortune associated with specific paired combinations of monetary outcomes and levels of probability. The task was divided in two conditions associated with separate blocks. In the “partial feedback” (PF) condition, the spinning arrow and the related outcome were presented for the selected wheel only. In the “complete feedback” (CF) condition the spinning arrows and the associated outcomes appeared both in the selected and rejected wheels (see Sect. 4.2 for a detailed description of the task). Based on previous evidence of altered WoF performance in pathological populations^6,29^, we predicted that AUD patients’ choice behaviour would reflect a) decreased integration of anticipated regret, and b) increased influence of near-miss outcomes, compared with healthy controls. We also explored possible group differences concerning other aspects of decision-making performance, including response time (RT), overall financial performance (W) and number of time-outs (TO; i.e. trials in which subjects did not respond within the available time-window). Information processing speed is indeed considered a possible marker of cognitive decline in several neurological diseases^32,33^, with a prominent role in age-related motor slowing as well^34^. Moreover, previous studies investigating alcohol-related decision-making impairments have shown increased choice latency as a possible marker of psychomotor slowing^35,36^, highlighting the “output” stage of decision-making as the most vulnerable to chronic alcohol consumption. Based on our previous findings, we thus predicted slower choice latencies in AUD patients compared with controls.

## Results

### WoF task performance and learning curve

**Figure 1:**
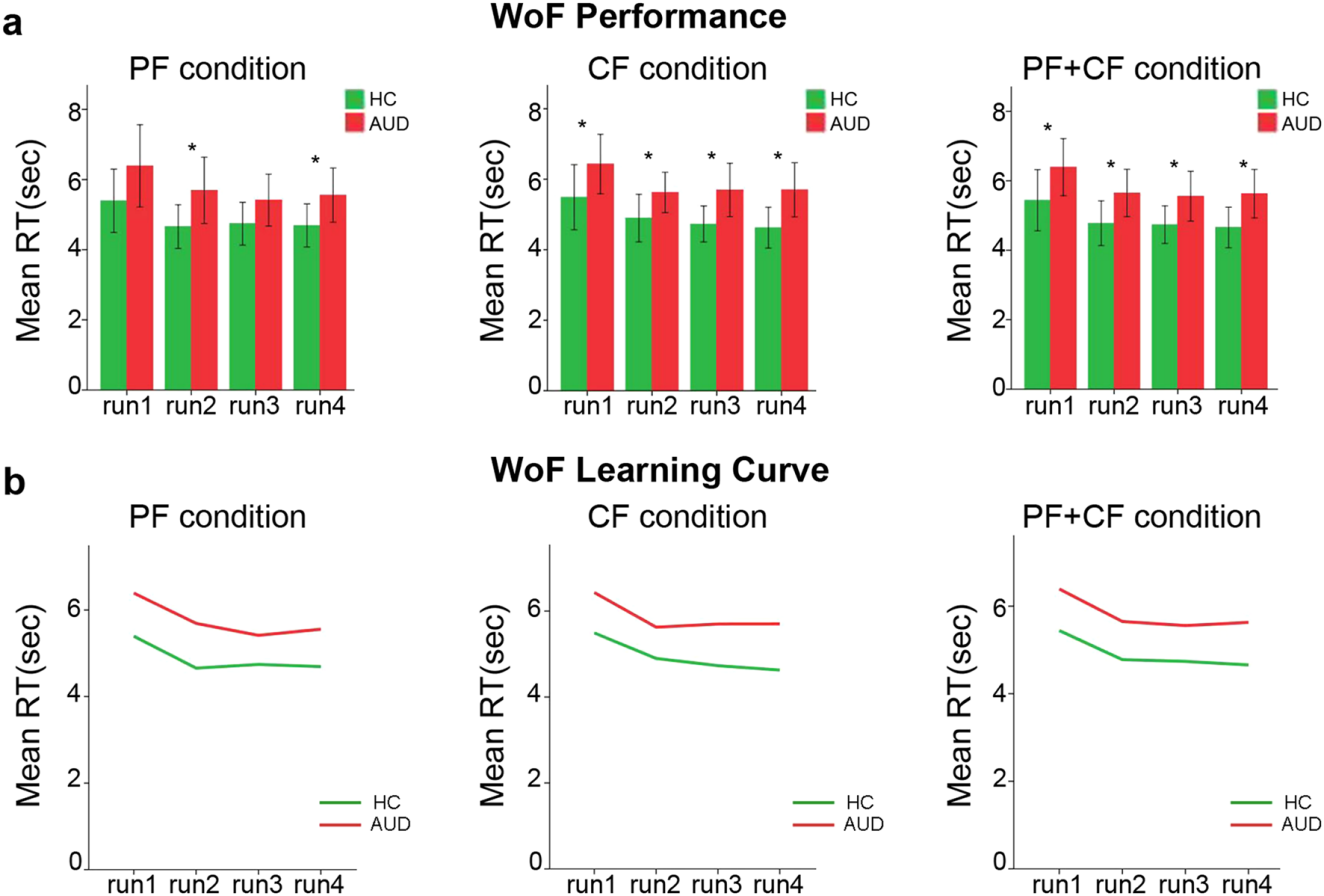
Task performance and learning curve. Panels (**a)** and (**b)** depict group differences in run-specific RTs and in learning curves, respectively, for partial- and complete-feedback conditions, or both.

Mann–Whitney U tests on PF and CF mean values highlighted, for both conditions, slower RTs in AUD patients compared with healthy controls (PF: p = 0.031r = − 0.328; CF: p = 0.012, r = − 0.397) (Table 1c). Instead, neither W (p = 0.339) or TO (p = 0.495) variables were significantly different across groups (Table1c). We found a positive correlation only between CF RTs and age (r = 0.262, p = 0.041). ANCOVA confirmed a significantly slower performance, in AUD patients, in CF trials after removing age effect (p = 0.045, η^2^ = 0.092; Table 1c). This finding was confirmed by additional analyses comparing performance across runs. AUD patients were slower than controls in all CF runs, but only in PF runs 2 (p < 0.015) and 4 (p < 0.38) (Table 2a, Fig. 1a). When testing the overall RTs regardless of condition, we confirmed AUD patients’ slower performance in all runs (Table 2a, Fig. 1a).

**Table 1:** Demographic and clinical variables.

	AUD (mean, ± SD) N = 26	HC (mean, ± SD) N = 19	DF	T/chi^2^*	p-value	
**a. Demographic variables**						
Sex (female/male)	10/16	8/11	1	0.061*	0.805	
Age	46.5 (± 8.25)	45.105 (± 8.69)	43	− 0.548	0.548	
Education (years)	10.88 (± 3.51)	10.63 (± 3.06)	43	− 0.252	0.802	

	AUD (mean, ± SD) N = 26	AUD – female (mean, ± SD) N = 10	AUD – male (mean, ± SD) N = 16	U	p-value	
**b. Alcohol use variables**						
Disease Duration (years)	10.77 (± 6.78)	11.78 (± 6.72)	10.14 (± 6.96)	67	0.492	
Daily Alcohol Use	15.42 (± 7.93)	14.85 (± 5.59)	15.78 (± 9.25)	− 76	0.853	
Abstinence (day)	14.27 (± 3.91)	13.50 (± 3.31)	14.75 (± 4.28)	65	0.452	

Partial feedback condition	AUD (mean, ± SD) N = 26	HC (mean, ± SD) N = 19	DF	U	p-value	r
**(c) WoF group comparison (Mann–Whitney)**						
RT (ms)	5,653.54 (± 1765.94)	4,872.31 (± 1,327.22)	43	166	**0.031**	− 0.328
W	55.36 (± 16.92)	53.98 (± 14.19)	43	229	0.339	− 0.073
To	1 (± 2.26)	0.58 (± 0.77)	43	246.5	0.495	− 0.002

Complete feedback condition	AUD (mean, ± SD) N = 26	HC (mean, ± SD) N = 19	DF	U	p-value	r
RT (ms)	57,800.04 (± 1,402.48)	4,930.25 (± 1,222.80)	43	149	0.012	− 0.397
W	66.96 (± 22.94)	72.79 (± 17.24)	43	200	0.140	0.190
To	0.68 (± 0.99)	0.63 (± 0.76)	43	221.5	0.254	0.103

	DF	F	p-value	η^2^		
**d. WoF group comparison controlling for Age (ANCOVA)**						
RT (partial feedback condition)	1.42	2.292	0.138	0.052		
RT (complete feedback condition)	1.42	4.262	**0.045**	0.092		

Section (a) reports demographic variables concerning gender, age and education for both AUD patients and healthy controls. Section (b) reports clinical information about alcohol use history and daily dose in AUD patients. Section (c) reports information about partial and complete feedback conditions (mean, standard deviation), concerning response time, gain and time-out variables obtained at the WoF task for AUD patients and healthy controls. Section (d) reports information about group differences in RT, corrected for the effect of age (ANCOVA). (*) indicates results from chi square test; all other analyses of group differences are based on non-parametric Mann–Whitney U tests for independent samples.

PF = partial feedback; CF = complete feedback; AUD = AUD patients; HC = healthy controls; SD = standard deviation; DF = degree of freedom; T = Student’s t-test; chi^2^ = chi square test; U = Mann–Whitney U Test; r = Rank biserial correlation; η^2^ = partial eta squared; FDR = False Discovery Rate adjustment applied on raw p-values.

Bold values denote statistical significance at the p < 0.05 level.

**Table 2:** Performance analysis.

	AUD (mean, ± SD) N = 26	HC (mean, ± SD) N = 19	U	p-value	r				
**a. RT group comparison**									
PF condition									
Run 1	6,390.13 (± 2,899.26)	5,395.01 (± 1871.74)	192	0.103	− 0,223				
Run 2	5,690.02 (± 2,338.19)	4,657.01 (± 1,292.31)	153	**0.015**	− 0.381				
Run 3	5,414.42 (± 1828.43)	4,741.81 (± 1,263.20)	192	0.103	− 0.223				
Run 4	5,555.93 (± 1915.30)	4,690.32 (± 1,277.79)	170	**0.038**	− 0.312				
CF condition									
Run 1	6,432.83 (± 2087.83)	5,490.92 (± 1916.46)	168	**0.034**	− 0.320				
Run 2	5,624.74 (± 1,421.98)	4,897.60 (± 1,400.54)	151	**0.013**	− 0.389				
Run 3	5,698.05 (± 1875.40)	4,729.53 (± 1,057.46)	161	**0.024**	− 0.348				
Run 4	5,701.78 (± 1901.15)	4,626.46 (± 1,207.30)	166	**0.031**	− 0.328				
PF + CF conditions									
Run 1	6,391.99 (± 2036.10)	5,440.88 (± 1,830.93)	169	**0.036**	− 0.361				
Run 2	5,649.65 (± 1687.29)	4,737.23 (± 1,338.20)	154	**0.016**	− 0.377				
Run 3	5,555.91 (± 1775.22)	4,737.48 (± 1,122.26)	175	**0.049**	− 0.291				
Run 4	5,629.13 (± 1729.84)	4,657.61 (± 1,213.96)	169	**0.036**	− 0.316				

	PF + CF		PF		CF				
	χ^2^	p-value	χ^2^	p-value	χ^2^	p-value			
**b. Friedman Test**									
AUD	12.60	**0.006**	7.062	0.070	5.862	0.119			
HC	7.737	0.052	10.137	**0.017**	9.379	**0.025**			

	PF + CF (AUD)			PF (AUD)			CF (AUD)		
	Z	p-value	r	Z	p-value	r	Z	p-value	r
**c. Wilcoxon Test**									
Run1-Run2	− 3.187	**0.001**	0.715	− 2.400	**0.016**	0.538	− 1.968	**0.049**	0.442
Run2-Run3	− 0.724	0.469	0.162	− 0.495	0.620	0.111	− 0.851	0.395	0.191
Run3-Run4	− 0.292	0.770	0.066	− 0.013	0.990	0.003	− 0.470	0.638	0.105

	PF + CF (HC)			PF (HC)			CF(HC)		
	Z	p-value	r	Z	p-value	r	Z	p-value	r
Run1-Run2	− 2.777	**0.005**	0.726	− 2.817	**0.005**	0.737	− 2.294	**0.022**	0.600
Run2-Run3	− 0.201	0.841	0.053	− 0.765	0.445	− 0.200	− 1.046	0.295	0.274
Run3-Run4	− 0.523	0.601	0.137	− 0.080	0.936	0.021	− 0.402	0.687	0.105

Section (a) reports data on run-specific group differences for partial- and complete-feedback conditions, or both (non-parametric Mann-Whitney U test). Section (b) reports information about RT differences across the 4 experimental runs. Section (c) reports the results of Wilcoxon tests aimed to unveil learning abilities. PF=partial feedback; CF=complete feedback; AUD=AUD patients; HC=healthy controls; SD=standard deviation; χ^2^= Friedman test; Z=Wilcoxon Test; r=Rank biserial correlation.

Bold values denote statistical significance at the p < 0.05 level.

Although we observed significant RT differences across runs in both groups (Table 2b), the analyses on learning curves showed fastest task performance at run 2 in the whole sample (AUD: p = 0.001, r = 0.715; HC: p = 0.005, r = 0.726), suggesting no group difference in the amount of time needed to stabilize performance (Table 2c, Fig. 1b).

### Choice behaviour

We tested two models of choice, incorporating the effect of different choice variables on decision-making behaviour. In the former, we modelled the effects of anticipating disappointment (d) and regret (r), alongside the maximization of expected value (e), under the assumption that individuals should aim to maximize EV while also learning to avoid the negative feelings associated with disappointment and/or regret (see details in^8^). The results of this model showed that, for both groups, choices in the PF condition were significantly modulated only by the maximization of expected value. In the CF condition, instead, healthy controls displayed a significant modulation by anticipated disappointment (β = 0.0000283, p < 0.036) but not regret (β = 0.0019097, p < 0.087), while neither variables were associated with significant effects in AUD patients (Table 3).

**Table 3:** Choice behaviour at the WoF task: Model 1.

	HC					AUD			
	Coeff	Std Error	z	p-value		Coeff	Std Error	z	p-value
Partial feedback condition									
e	0.0001851	0.0000116	15.96	** < 0.0001**	e	0.0001651	0.00000884	18.67	** < 0.0001**
d	− 0.00000227	0.00000791	− 0.29	< 0.402	d	− 0.000000816	0.00000652	− 0.13	< 0.901
Complete feedback condition									
e	0.0001549	0.0000195	7.93	** < 0.0001**	e	0.0001818	0.0000174	10.46	** < 0.0001**
d	0.0000283	0.0000135	2.09	** < 0.036**	d	0.00000749	0.0000114	0.66	< 0.511
r	0.0019097	0.0011156	1.71	< 0.087	r	− 0.0002403	0.0009492	− 0.25	< 0.800

The table reports the results of a model of choice integrating the effects of anticipated disappointment (d) and regret (r) in addition to the maximization of expected value (e).

Bold values denote statistical significance at the p < 0.05 level.

The second model aimed to investigate the modulation of choice behaviour by the affective experience associated with a near-miss outcome (NM) in the previous trial, in addition to the maximization of expected value (e). The results revealed group-specific modulations of choice behaviour depending on the experimental condition (Table 4). Choices in the PF condition were driven only by the maximization of expected value in healthy controls (β = 0.0008, p < 0.0001), but also by previous experiences of near-miss outcomes in AUD patients (β = 0.28, p < 0.006). The contrary was true for the CF condition, in which choices were guided only by expected value in AUD patients (β = 0.000191, p < 0.0001), and also by past experiences of near-miss outcomes on the unchosen gamble in healthy controls (β = − 0.28, p < 0.023).

**Table 4:** Choice behaviour at the WoF task: Model 2.

	HC					AUD			
	Coeff	Std.Error	Z	p-value		Coeff	Std.Error	Z	p-value
Partial feedback condition									
e	0.0007845	0.0000112	16.4	** < 0.0001**	**e**	0.0001717	8.98E−06	19.13	** < 0.0001**
NM_C	0.0268493	0.1215746	0.22	< 0.825	**NM_C**	0.279621	0.1018746	2.74	** < 0.006**
Complete feedback condition									
e	0.0001987	0.0000129	15.38	** < 0.0001**	**e**	0.000191	0.0000108	17.62	** < 0.0001**
NM_C	0.1536473	0.1362582	1.13	< 0.259	**NM_C**	0.1549444	0.1154768	1.34	< 0.180
NM_UC	− 0.2819862	0.1239845	−2.27	** < 0.023**	**NM_UC**	− 0.1460421	0.2498032	0.21	< 0.835

The table reports the results of a model of choice integrating the effects of previous experiences of near miss outcome associated with the chosen (NM_C) and unchosen (NM_UC) gambles, in addition to the maximization of expected value (e).

Bold values denote statistical significance at the p < 0.05 level.

## Discussion

Alcohol use disorder is one of the most prevalent psychiatric conditions worldwide. The Diagnostic and Statistical Manual of Mental Disorders—fifth edition—defined AUD as a chronic relapsing condition characterized by excessive alcohol consumption despite its devastating consequences on individuals’ physical, social and cognitive functioning^47^. In particular, the revised diagnostic criteria for AUD highlighted the impairment in behavioural control, an important high-order cognitive function implicated also in decision-making process.

Alcohol-related decision-making impairments are increasingly investigated by focusing on possible alterations of the computational facets of choice behaviour^25,26^. While previous studies have highlighted altered reinforcement learning as a core component of the typical vicious circle linking craving, immediate rewarding effects and long-term negative consequences, it is still unknown whether such impairments also involve choice-related, cognitively mediated, emotions such as regret and relief, which have been shown to support adaptive behavioural learning in healthy individuals^8^. We addressed this issue with a cognitively demanding decision-making task incorporating several choice variables, such as expected value, satisfaction for a gain or disappointment for a loss, complex emotions, such as regret or relief, and the influence of previously experienced near-miss outcomes.

AUD patients and healthy controls did not differ in terms of overall payoff, number of time-outs or learning curve throughout the task, with RTs stabilizing at the second run in both groups. AUD patients, however, were significantly slower than healthy controls in making their choices both in PF and CF conditions. In line with the role played by information processing speed in higher-order cognitive tasks^37^, these results confirm previous reports of alcohol-related increased deliberation time^35,36,38^, likely reflecting a generalized executive impairment extending to the output stages of decision-making^39–42^. However, when controlling for possible age effect in CF trials we observed significantly longer RTs in AUD patients compared with healthy controls. The results of behavioural modelling allowed assessing whether AUD patients’ sensitivity to this condition, over and beyond possible age effect, reflects a defective incorporation of anticipated negative emotions in choice behaviour.

In line with our hypothesis, AUD patients chose by maximizing expected value, but failed to minimize both disappointment and regret. Unlike healthy controls, who displayed a significant anticipation of disappointment, they thus neglected the affective consequences of their choices when evaluating gambles. This impairment might contribute to AUD patients’ behavioural alterations in everyday life. The ability to anticipate negative emotions is indeed considered a powerful motivator to change behavioural strategies in order to reach better outcomes^43^ associated with healthy behaviours. The defective estimation, and/or incorporation, of “reward” and “fictive” prediction errors might thus represent the computational basis of AUD patients’ neglect for the affective consequences of their choices. Since the associated emotions of disappointment and regret are considered to enhance adaptive behavioural learning from past experiences, driving motivated behaviour away from risk, the observed impairment is thus likely to promote patients’ inability to learn from the negative consequences of chronic alcohol consumption, and thus the maintenance of AUD. Unlike previous studies, we did not observe a significant minimization of anticipated regret in healthy controls^6,8^. This negative finding might be explained by the higher age mean and standard deviation in our sample (Table 1) compared with previous studies on regret processing^7,29^.

The hypothesis of a defective incorporation of affective information in AUD patients’ evaluative processes was supported by a second model of choice, testing the effect of near-miss outcomes alongside expected value. While both groups chose by maximizing expected value, they displayed different modulations by the emotional experience associated with near-miss outcomes, biasing only the PF condition in AUD patients, and only the CF condition in healthy controls. To date, near-miss outcomes have been interpreted either as frustrating events reinforcing maladaptive behaviours to diminish the associated negative emotional state^45^, or as positive reinforcers mentally represented as actual appetitive outcomes^46^. The former interpretation fits with the role played by negative reinforcement in AUD, i.e., by the need to escape the aversive state associated with the craving for alcohol^27^. In either case, however, near-miss outcomes are known to exert their effect by potentiating the affective load attached to *what might have been* under a different fate in the PF condition, or a different choice in the CF one. Regardless of a specific interpretation, this finding confirms that the defective integration of information concerning the unselected option represents a consistent trait of AUD patients’ decision-making processes, decreasing the chances of learning from previous negative experiences.

There are limitations to this study. First, the accurate case–control matching for demographic variables and our stringent inclusion criteria resulted in a small-to-moderate sample size. Moreover, the lack of specific measures of information processing speed did not allow establishing a causal connection between the executive and computational impairments displayed by AUD patients. The present results should thus be considered as preliminary evidence in need of further support from studies including larger samples and additional neuro-cognitive variables. Finally, here we focused our attention only on the computational aspects of cognitive-based emotions of regret and relief, disappointment and satisfaction, neglecting the role of individual differences in perception and interpretation of such complex affective states. Further investigations are needed in order to assess the relationships between the impaired ability of AUD patients in implementing cognitive-based emotions during decision-making and their relative subjective feelings. To the best of our knowledge, however, this is the first study investigating AUD patients’ decision-making performance by modelling the anticipation of complex emotions resulting from counterfactual thinking^31^. Although preliminary and in need of further supporting evidence, our results highlight the defective implementation, and thus avoidance, of disappointment and regret as a component of AUD patients’ alterations in learning from negative experiences. Future research might build on these results by addressing gender differences, the neural bases of these alterations, as well as the development and assessment of treatment protocols specifically focused on the implementation of emotional experiences in choice-related behaviours.

## Materials and methods

### Participants

Twenty-six adult AUD patients (10 females; mean age: 46.50 years ± 8.25; range: 29–64; mean education: 10.88 years ± 3.51) and 19 age- and education-matched healthy control subjects (8 females; mean age: 45.11 years ± 8.69; range: 27–57; mean education: 10.63 years ± 3.05) participated in the study. A chi-square test confirmed that the distribution of males and females was not significantly different across AUD patients and healthy controls (p = 0.805). AUD patients were enrolled from the Functional Rehabilitation Unit of ICS Maugeri-Pavia (Italy), and healthy controls were recruited via local advertisement. There was no significant demographic difference between AUD patients and healthy controls (Table 1). Average disease duration in AUD patients ranged from 1 to 26 years (mean: 10.77 years ± 6.78). Inclusion criteria for AUD patients were: 1) age between 20 and 60 years; 2) a diagnosis of alcohol dependence according to DSM-V diagnostic criteria. Exclusion criteria for both AUD patients and control subjects were: (1) presence or history of neurological or psychiatric disorders other than AUD, or any comorbid disorder except for nicotine dependence; (2) family history of neurological or psychiatric disorders; (3) current use of any psychotropic substance or medication; (4) past brain injury or loss of consciousness; (5) major medical disorders (e.g. kidney or liver diseases, severe diabetes and/or malnutrition); (6) inability to undergo the neuropsychological assessment. Healthy controls were also excluded in case of presence or history of alcohol abuse. AUD patients joined the experimental protocol after being detoxified for at least 10 days, via medically supported standard treatments. However, they had ceased benzodiazepine treatment at least 8 days before scanning. Healthy participants were at least abstinent 10 days before scanning. All participants provided written informed consent to the experimental procedure, which was approved by the local Ethical Committee of ICS Maugeri-Pavia. The investigation was conducted in accordance with the latest version of the Declaration of Helsinki (see also^35,39–42^).

### Wheel of Fortune (WoF) Task

**Figure 2:**
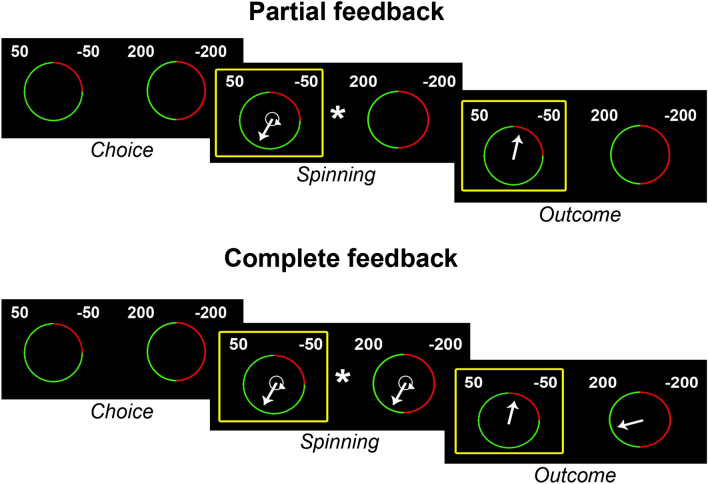
Wheel of fortune (WoF) task. Subjects are repeatedly asked to choose between two gambles, depicted as wheels of fortune associated with specific paired combinations of monetary outcomes (200, 50, − 50 and − 200) and levels of probability represented by the size of the green/red sectors (20–80, 50–50 and 80–20). The possible counterfactual combinations of wins and losses result in four potential levels of regret and relief (± 100, ± 150, ± 250 and ± 400) when subjects are shown the outcomes of both the selected and unselected gambles (CF). In the PF condition, instead, only the outcome of the selected gamble is shown, thus resulting in satisfaction or disappointment for the best or worse outcome. The gambles were shown for 4.5 s, during which subjects could evaluate them and make a decision. Next, the appearance of an asterisk in the centre of the screen prompted them to choose a gamble, which was highlighted by a yellow contour. Two seconds after the appearance of the asterisk a white arrow appeared in a random position in both wheels (CF) or only in the selected wheel (PF). One second later, the arrow(s) started spinning clock-wise, with different and random initial speed(s), and stopped after exactly 4 s, indicating the final outcome(s), that remained on the screen for 3 s.

The WoF task is an experimental paradigm adapted from Mellers and colleagues^31^, previously used to investigate the contribution of choice-related emotions such as satisfaction/disappointment and relief/regret to decision-making under risk^6,8,29^. Subjects are repeatedly asked to choose between two gambles, depicted as wheels of fortune, on the left and right halves of the screen. Each wheel is divided in two sectors, i.e. green (left) and red (right), always associated with the best and worse outcomes, respectively (Fig. 2). In each gamble, the possible outcomes involve paired combinations of 200, 50, − 50 and − 200 (arbitrary units), associated with 3 different levels of probability (20–80, 50–50 and 80–20) represented by the size of the green/red sectors. Therefore, the possible counterfactual combinations of wins and losses result in four potential levels of regret and relief (± 100, ± 150, ± 250 and ± 400) when subjects are shown the outcomes of both the selected and unselected gambles (CF). Thus, if the computational process results in a positive outcome they will experience relief, otherwise regret. In this condition, indeed, they can evaluate not only the financial consequences of their decision, but also the outcome they might have obtained, if they had selected the alternative gamble. In the PF condition, instead, only the outcome of the selected gamble is shown, thus resulting in satisfaction or disappointment for the best or worse outcome, respectively, without a sense of personal responsibility.

In the present study, the gambles were shown for 4.5 s, during which subjects could evaluate them and make a decision. Next, the appearance of an asterisk in the centre of the screen prompted them to choose, by pressing one of two buttons on a keyboard with their right index or middle fingers. Subjects had 2 s to choose the gamble, otherwise they received an “out of time” message, and a new trial started. Once selected, the chosen gamble was highlighted by a yellow contour, that would remain on the screen up to the end of the trial, and 2 s after the appearance of the asterisk a white arrow appeared in a random position in both wheels (CF) or only in the selected one (PF). One second later, the arrow(s) started spinning clock-wise, with different and random initial speed(s), and stopped after exactly 4 s, indicating the final outcome(s), that remained on the screen for 3 s. The spatial distance between the resting position of the spinning arrow and the boundary between the green and red sectors was used to code three possible values of “closeness” of near-miss outcomes (see below).

Each participant performed 192 trials overall, blocked in 4 runs of 24 trials per condition. The order of CF and PF blocks was counterbalanced across subjects. Because of the complexity of the task and in order to make participants confident with answer’s procedure, each subject performed a training session before to start.

### Definition of choice variables

We used the following variables to investigate possible group differences in task performance and choice behaviour: response time to select the gamble (RT); number of time-outs (TO); expected value (EV, i.e. the sum of the value of the two possible gamble outcomes, each weighted by the corresponding probability); gain (W, the value associated with the outcome); disappointment (d, the negative emotion associated with the difference between the actual and unobtained outcomes of the selected gamble); regret (r, negative emotion associated with the difference between the outcomes of the selected and rejected gambles); near-miss outcomes (NM; the degree of “closeness” of non-win outcomes).

For each variable we computed: a) the overall mean value in PF and CF conditions separately; b) the mean value in each of the 4 PF and CF runs; c) the mean value for each of the 4 runs regardless of the experimental condition.

### Analysis of choice performance

To evaluate task performance, we considered RT, TO and W variables as representative indexes of subjects’ ability to perform the WoF task. Thus, we first checked for possible group differences by means of Mann–Whitney non-parametric U tests on the overall PF and CF mean values, separately. We then checked for age and education effects by means of correlation analyses. For those variables showing both between-group differences and a significant effect of age and/or education, we ran an Analysis of Covariance (ANCOVA) to assess the stability of results after removing their effects. We applied a primary statistical threshold of p < 0.05, one-tailed due to a priori hypotheses of alcohol-related impairment^23^.

To investigate possible effects of chronic alcohol consumption on learning curves, we then explored group differences among runs. Namely, for the variables showing significant group differences in the previous analysis we performed additional Mann–Whitney non-parametric U tests on run-specific mean values regardless of condition, as well as PF and CF mean values separately. Based on the considerable executive load of the WoF task, we also assessed possible group differences in learning abilities in terms of time needed for task execution. To this purpose, we first applied the Friedman test within each group, to highlight significant RT differences along the four runs. We then performed post-hoc analyses (Wilcoxon signed-rank test) to identify the specific run at which each group reached the maximum (i.e. fastest) performance. For each statistical analysis we performed also effect size calculation, i.e. Glass rank biserial correlation for non-parametric tests and partial eta squared for ANCOVA. Statistical analyses were performed with SPSS (IBM Corp. Released 2015. IBM SPSS Statistics for Macintosh Version 23.0. Armonk, NY: IBM Corp.) and JASP (https://jasp-stats.org/).

### Analysis of choice behaviour

We applied regression analyses, using a panel logit procedure with an individual random effect, to unveil the contribution of different choice-related emotions to decision-making performance. The panel data analysis modelled each subject as unit, and each trial as time variable. The random-effects model was used as the default model, and the parameters were estimated by maximum likelihood. We tested two models of choice, incorporating the effect of different choice variables on decision-making behaviour.

In the former, we modelled the effects of anticipating disappointment (d) and regret (r), alongside the maximization of expected value (e) (see details in^8^). The probability of choosing gamble 1 is:

1 $$Pr\left({g}_{1st}\right)=1-Pr\left({g}_{2st}\right)=F\left[{d}_{st};{r}_{st}{;e}_{st}\right]$$

where *s* = subject, *t* = time and F[θ] denotes the function e^θ^/(1 + e^θ^). The variables *d* and *r*, as described in Eqs. 2 and 3, indicate the process of minimizing future disappointment and future regret, respectively; *e* indicates the result of maximizing expected values. *x*_*1*_ and *y*_*1*_ represent the better and worst outcome of gamble 1 (*g*_*1*_), and *x*_*2*_ and *y*_*2*_ represent the better and worst outcome of gamble 2 (*g*_*2*_). The probability of *x*_*1*_ is *p* and the probability of *y*_*1*_ is *1 – p*; the probability of *x*_*2*_ is *q*, and the probability of *y*_*2*_ is *1 – q*.

2 $$d=\left({y}_{2}-{x}_{2}\vee \left(1-q\right)\right)-\left({y}_{1}-{x}_{1}\vee \left(1-p\right)\right)$$

3 $$r=\left|{y}_{2}-{x}_{1}\right|-\left|{y}_{1}-{x}_{2}\right|$$

4 $$e=EV\left({g}_{1}\right)-EV\left({g}_{2}\right)=\left({px}_{1}+\left(1-p\right){y}_{1}\right)-\left({qx}_{2}+\left(1-q\right){y}_{2}\right)$$

In the CF condition, the optimal behaviour depends on the subject’s ability to minimize disappointment (d; Eq. 2) and regret (r; Eq. 3), while maximizing expected value (e; Eq. 4). In the PF condition, instead, subjects can only integrate in their evaluative process the effect of anticipated disappointment and maximization of expected values (see details in^8^).

The second model aimed to investigate the modulation of choice behaviour by the affective experience associated with a near-miss outcome (NM) in the previous trial, in addition to the maximization of expected value (e). Therefore, choice behaviour depends a) both on the maximization of expected value (Eq. 4) and near-miss outcome of both gambles in the CF condition; b) only on expected value and near-miss of the chosen gamble in the PF condition. The probability of choosing *g*_*1*_ is:

$$Pr\left({g}_{1st}\right)=1-Pr\left({g}_{2st}\right)=F\left[{NM}_{st}{;e}_{st}\right]$$

where the NM regressor included three possible values, depending on the proportional distance between the resting position of the spinning arrow and the boundaries between the best and worse gamble outcomes. Therefore, the participants could experience a "clear" outcome, when the arrow stopped in the central area of the best or worse gamble segments; a full near-miss outcome, when it stopped very close to the boundary between them; and a partial near-miss outcome, when it stopped in an approximately intermediate area between the former positions.

The analysis of choice behaviour was carried out with STATA (StataCorp. 2019. *Stata Statistical Software: Release 13*. College Station, TX: StataCorp LLC.).
